# OnabotulinumToxinA Reduces Pharmacological Burden in Chronic Migraine Patients: A Two-Center Prospective Cohort Study

**DOI:** 10.3390/toxins18030143

**Published:** 2026-03-14

**Authors:** Danilo Antonio Montisano, Alessandra Parisi, Alberto Raggi, Claudia Altamura, Luigi D’Onofrio, Marilena Marcosano, Luisa Fofi, Alessia Marcassoli, Fabrizio Vernieri, Licia Grazzi

**Affiliations:** 1 Headache Center, Fondazione IRCCS Istituto Neurologico Carlo Besta, 20131 Milano, Italylicia.grazzi@istituto-besta.it (L.G.); 2 Neurology, Public Health and Disability Unit, Fondazione IRCCS Istituto Neurologico Carlo Besta, 20133 Milano, Italy; 3 Headache and Neurosonology Unit, Fondazione Policlinico Campus Bio-Medico, Università Campus Bio-Medico Di Roma, 00128 Rome, Italy

**Keywords:** chronicmigraine, OnabotulinumtoxinA, preventive, pharmacological, burden

## Abstract

Background: Chronic migraine (CM) is a highly disabling and difficult-to-manage condition with a high pharmacological and economic burden. OnabotulinumtoxinA (BTx) was the first treatment specifically approved for CM. The main aim of this study was to assess whether the initiation of BTx is associated with discontinuation of previously prescribed preventive therapies. Methods: This study was a prospective cohort investigation conducted in two headache centers: Carlo Besta (Milan) and Policlinico Campus Bio-Medico (Rome). We included patients with CM and previous oral preventive treatments initiating BTx. We analyzed persistence with preventive therapies over 12 months of follow-up and evaluated the conversion rate from chronic to episodic migraine (EM), along with change in migraine days, symptomatic intake, and HIT-6. Results: A total of 95 patients were included in the main analysis, showing a discontinuation of treatment in 28.4% of patients at 12 months. In the exploratory analysis, a CM to EM conversion rate of 58.9% was achieved at 12 months; meanwhile, HIT-6, migraine days, and symptomatic intake showed a sizeable improvement. Conclusion: Treatment with BTx was associated with a reduction in drug burden at 12 months and a CM to EM conversion rate of almost 60% at 12 months, also contributing to a reduction in the economic burden of the disease.

## 1. Introduction

Migraine is a highly prevalent neurological disorder, with a prevalence of 15.2% globally (18.9% in women and 11.4% among men), which poses a significant burden, that becomes even more relevant in its chronic form. In fact, all over the world, it is ranked among the leading causes of health loss as measured by the GBD, especially among women aged 15 to 49 years old [1]. It is characterized by recurrent disabling headache attacks accompanied by a wide range of neurological symptoms, such as photophobia, phonophobia, nausea, and vertigo, and almost 30% of patients’ pain is accompanied or preceded by migraine aura, most usually represented by transient visual disturbance. Besides the clinical core symptoms, migraine is frequently associated with several comorbidities, among which are psychiatric conditions such as anxiety and depression, that negatively affect the patient’s quality of life [1]. Chronic migraine (CM), defined according to the ICHD3 [2] as a headache occurring on at least 15 days per month, of which at least 8 are with a migraine phenotype, is associated with various clinical and management issues, resulting in high direct and indirect costs and in a drastic reduction in patients’ quality of life [1,3,4]. CM burden is even more pronounced, with high levels of disability, reduced quality of life, increased healthcare utilization, and significant socioeconomic impact, with important work and social consequences, resulting in significant direct and indirect costs. It is also associated with a higher prevalence of psychiatric comorbidities (such as depression and anxiety), pain disorders, sleep disturbances, and increased cardiovascular risk [1,3,5].

Due to suboptimal control, patients often present a high use of symptomatic drugs and may even develop medication overuse headache (MOH), which is defined as more than 10 days per month of utilization of opioids, triptans, or combination analgesics, or more than 15 days per month of simple analgesic or non-steroidal anti-inflammatory (NSAID) medications. In CM, approximately 50% of patients overuse acute medications [5]. So, pharmacological management often requires different therapeutic approaches, and polytherapy is therefore common, which in turn might increase the risk of developing drug-induced side effects [6].

The first treatment specifically approved for CM was OnabotulinumtoxinA (BTx), approved by the FDA in 2009 upon pivotal studies that demonstrated a significant clinical improvement, with a mean reduction of 8.4 days of headache [7,8,9]. BTx acts by inhibiting the release of pain-mediating neurotransmitters (such as CGRP, substance P, and glutamate) from peripheral sensory nerve terminals, thereby reducing peripheral and central sensitization within the trigeminovascular system through the blocking of synaptic vesicle fusion at the pre-synaptic level. More specifically, BTx disrupts the release of neurotransmitters from the synaptic membrane by inhibiting the soluble N-ethylmaleimide-sensitive fusion-attachment protein receptor (SNARE) complex, formed by the proteins synaptobrevin and syntaxin, and synaptosomal-associated protein 25 (SNAP-25) [10]. Over the years, real-world evidence has also shown that BTx treatment is truly effective, reducing several significant aspects of the clinical spectrum, such as allodynia and the overall impact of headaches [11,12]. In addition to BTx, only Topiramate, an antiepileptic drug (AED), has been specifically tested for CM and provides a clear indication for this condition, but it has several limitations, such as challenging tolerability and, of particular note, a teratogenic effect on fetuses, that often restrict its use [1].

However, with those specific approaches, the whole burden of CM is also still high. Part of this high burden is pharmacological, namely the joint use of several compounds both for acute and prophylactic use, which might increase the risk of side effects [13,14] but also increase the cost of CM. It is therefore of great importance to address the degree to which the choice of a specific compound for prevention might reduce the overall number of prophylactic lines.

The primary aim of this study was to investigate whether the initiation of BTx was associated with the discontinuation of other previous ongoing prophylactic treatments in patients with CM.

## 2. Results

A total of 95 patients (87 females, corresponding to 91.6%) were included; the median age was 53 ± 11.6 (range: 18–76); and 51 patients reported comorbidities, mostly psychiatric traits (anxiety and depression, 20 patients), hypertension (14 patients), and endocrinological diseases (mostly dysthyroidism, 11 patients). On average, patients previously failed 3 ± 1.7 (range: 1–9) preventive therapies. As baseline preventive treatments, the most represented class was antidepressants, followed by antiepileptics and beta blockers. Table 1 reports baseline description variables.

**Figure 1:**
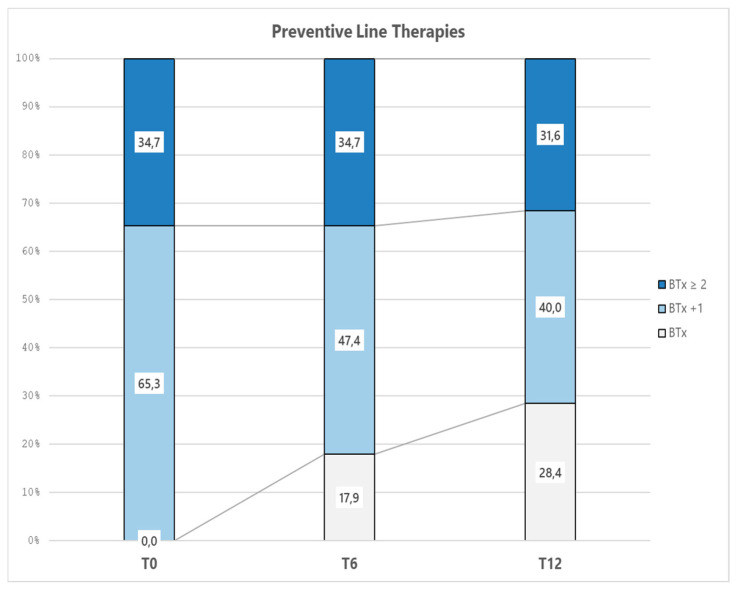
Representation of the proportion of preventive line therapy groups. BTx = only BTx treatment; BTx+1 = BTx and one additional preventive line; BTx+2 = BTx and two or more additional preventive lines.

Table 2 and Figure 1 report the frequency and percentage over the study period for treatment group, diagnosis, and diagnosis change. With regard to our primary endpoint, 17.9% and 28.4% of the patients were on BTx only at 6 and 12 months, respectively, and 57.9% and 58.9% matched the diagnosis of EM (at 6 and 12 months, respectively). Regarding diagnosis change, a minor part of the sample (28.4%) remained chronic, whereas some kind of improvement was shown in the rest of the sample. It must be noted that almost half of the sample maintained a stable EM diagnosis between 6 and 12 months.

Regarding the other groups, the proportion of patients receiving BTx in combination with one additional prophylactic line (BTx+1) decreased from 65.3% at baseline to 47.4% and 40.0% at 6 and 12 months, respectively. Similarly, the proportion of patients receiving BTx in combination with two or more additional preventive lines (BTx+2) remained stable from baseline to 6 months (34.7%) and decreased to 31.6% at 12 months. The reductions observed in both groups over time suggest a progressive optimization of the therapeutic approach, with an increasing proportion of patients achieving adequate disease control with fewer prophylactic lines.

**Figure 2:**
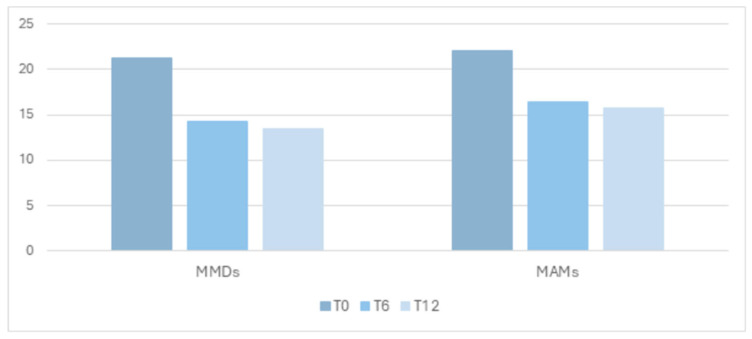
Representation of clinical variations in MMDs, MAMs, and HIT-6 at 0, 6, and 12 months. Notes: MMDs = monthly migraine days, MAMs = monthly acute medications, HIT-6 = six-item Headache Impact Test.

All clinical variables improved over time after BTx initiation (Table 3 and Figure 2). In particular, the amount of non-BTx treatments was significantly reduced, and such a reduction was accompanied by a decrease in headache frequency and medication intake. The frequency of migraine attacks (MMDs) saw a progressive decrease in mean frequency from baseline (M ± SD) 21.2 ± 6.3, to 6 months 14.3 ± 8.5, and to 12 months 13.5 ± 7.6. Drug intake (MAMs) showed a progressive decrease in mean frequency from baseline 22.1 ± 13.4, to 6 months 16.4 ± 16.4, and to 12 months 15.8 ± 17.5 follow-up. Even HIT-6 showed a favorable trajectory, from baseline 66.7 ± 6.3, to 12 months follow-up 63.8 ± 7.0. Scores for MMD significantly decreased from baseline to 6 months (Z = −6.6, *p* < 0.001) and from baseline to 12 months (Z = −7.1, *p* < 0.001). The comparison between 6 and 12 months was not statistically significant, indicating stability over time. Similarly, MAM decreased significantly from baseline to 6 months (Z = −3.9, *p* < 0.001) and from baseline to 12 months (Z = −4.8, *p* < 0.001). The comparison between 6 and 12 months was not statistically significant.

When specific preventive treatments were considered, no specific pattern in reduction was observed: 15% for antidepressants, 30% for antiepileptics, 26% for beta blockers, 17% for sartans, and 37% for other medications. The change in HIT-6 (calculated between baseline and 12 months only) was −2.9 points (95%CI: −1.3 to −4.5).

## 3. Discussion

Our results show that starting BTx therapy leads to an overall reduction in pharmacological burden in patients with CM. After 12 months, almost one third of patients (28.4%) switched to BTx only, and 58.9% of patients moved from CM to EM. In addition to this, a sizeable reduction in MMDs, MAMs, and HIT-6 score was observed.

These results are highly significant in a condition that often requires several therapeutic attempts and simultaneous treatment with different active compounds, also considering that these patients have a long history of failed treatments and experience a considerable level of disability [15]. BTx treatment also has the advantage of providing clinical improvement while reducing the overall use of acute and preventive medication, tied with a minimal systemic impact on the patient. This is of great importance for this population of severe patients, who often experience significant medical comorbidities and are difficult to treat [16]. Reducing polytherapy is an important target of modern medicine, as polytherapy is associated with a higher risk of adverse events, even though drug-to-drug interaction, which reduces patients’ adherence to treatments and leads to discontinuation [17].

A sizeable improvement was achieved for MMD and MAM: the first was reduced by 6.9 days at 6 months and 7.7 days at 12 months, whereas the second was reduced by 5.7 and 6.3 intakes at 6 and 12 months, respectively. These results are in line with pivotal findings [7,8,9]. The role of BTx in CM prevention is well recognized and has been confirmed by various scientific findings in the literature in recent years. Aside from pivotal studies, even real-world (RW) data broadly support its utility. In a meta-analysis of the RW literature, at 52 weeks follow-up, an overall reduction in headache days of mean (IC) −10.3 (−14.9, −5.73) and of days of acute headache pain medication per month of −7.4 (−13.04, −1.77) have been reported, data corroborating the efficacy observed in the pivotal trials even in a long-term and real-life scenario [18].

Moreover, a recent umbrella review across 14 systematic reviews with meta-analyses showed favorable effects of BTx on headache frequencies, severity, and acute medication use. Though its effect was less pronounced than that of topiramate anti-CGRP mAbs (e.g., galcanezumab and fremanezumab), it displayed superior tolerability compared to topiramate, similar to anti-CGRP mAbs [19].

The reduction in disease burden is not only observable from a clinical perspective but also when considering disease cost. In fact, the cost associated with CM is three- to fourfold that associated with EM, mostly driven by indirect medical cost [1]. We observed a CM to EM conversion rate of 57.9% at 6 months and of 58.9% at 12 months, similar to what was observed in the CaMEO study (49.9%) [20]. These results extend beyond clinical improvement, pointing to a more complex economic burden of disease. Indeed, as shown by Raggi et al., switching to EM could mean saving around 530€/month per patient [21]. In the first 6 months of treatment, 55 patients out of 95 patients (57.9%) converted from CM to EM status, and by the 12th month, 13 out of a total of 56 patients, who were still in CM, achieved EM status. On the other hand, 12 patients progressed again to CM, but 45 were stable in EM.

It should be noted that the rising proportion of patients who improved through the treatment period and discontinued previously prescribed prophylaxis (both at 6 and 12 months) may be partly linked to the long-term neuromodulation effect of BTx. In fact, BTx exerts its effect on the central mechanisms of pain from the periphery by acting on central sensitization, which is a core mechanism for the maintenance of CM. Our result, pointing to a cumulative effect at both 6 and 12 months, is in line with previous evidence showing the cumulative efficacy of BTx over the first 12 months of treatment [22,23]. This mechanism of action may prove to be fundamental in interrupting chronicity and be at the base of evaluating therapeutic add-on with other innovative anti-CGRP drugs. In fact, dual therapeutic action, between the modulation of C fibers and a-delta fibers, could be particularly beneficial in populations with a higher disease burden, and some data are already available [24]. In line with this observation, the lowest reduction by class of preventive treatments was observed for antidepressants (−15% only), which is, in any case, not irrelevant considering that 20 out of 95 patients (i.e., 21%) had psychiatric comorbidities.

A proportion of patients, corresponding to approximately 32% of our sample, were on polytherapy at baseline and remained on polytherapy also at 12 months. This group could represent a more complex group of patients for concomitant medical issues; in fact, those who were prescribed BTx and two or more additional preventive lines were also those with the highest comorbidity rate (73.3% vs. 44.6% of those prescribed BTx only or BTx and one additional preventive line). Although this could be a speculative explanation, it is reasonable to presume that these patients are on polytherapy not only for unsatisfactory migraine control but also for treating comorbidities. A similar conclusion was presented by the PREVENAC study results [25], in which comorbidities were among the factors associated with continued use of concomitant preventives (such as insomnia and hypertension). Similarly, Fofi et al. [26] showed a discontinuation of previous preventive treatment in patients starting anti-CGRP mAbs after 12 months in 109 out of 195 patients (44.1%), and also highlighted that their experiences of comorbidities were associated with a high degree of preventive treatment retention.

Our results corroborate the previous evidence from Alpuente and colleagues [27], who, in a retrospective study on 542 patients, reported a 41.6% discontinuation rate of all oral prophylactic treatments, higher than what was found in our study (28.4%). This difference could be related to a different observation period between the two studies. In fact, we followed patients prospectively for up to 12 months, which is a typical full cycle of BTx treatment, whereas Alpulente and colleagues reported a retrospective evaluation covering the entire clinical history of patients, with an average of 5 years or more of BTx treatment. This further reinforces the hypothesis that prolonged treatment with BTx might be associated with an increased cumulative benefit.

This broad view of evaluating CM patient improvement is not new [28]; in fact, both the CM to EM conversion rate and the reduction in oral preventive therapies could help to provide important clinical data in a population where the standard 50% improvement in terms of headache days could not be so meaningful.

Our study has some limitations that should be considered in the interpretation of the results: (i) the small sample size; (ii) the observational design of the study, which did not allow a standard discontinuation of prophylaxis approach among clinicians at the various centers, with the risk of obtaining heterogeneous results; (iii) the lack of information on the possible occurrence of new comorbidities; (iv) the lack of other PROMs in addition to HIT-6 to better describe the relations between clinical benefits and patients’ perspective; (v) the absence of a control group limits the attribution of results to the therapeutic intervention alone.

## 4. Conclusions

In conclusion, our prospective cohort study on patients with CM treated with BTx showed a cumulative reduction in the pharmacological burden at 6 and 12 months in this complex population: 28.4% switched to BTx only, and a sizeable reduction in MAMs and MMDs was observed as well. At the same time, a conversion rate from CM to EM of almost 60% was observed throughout the 12-month study period. Both data may provide a new measure of improvement in the overall burden of CM.

## 5. Materials and Methods

### 5.1. Study Design and Participants

This was a multicenter, retrospective analysis of a prospective observational cohort study, conducted among two tertiary headache centers across Italy: Fondazione Istituto Neurologico C. Besta, in Milan, and Fondazione Policlinico Campus Bio-Medico, in Rome. The observation period was twelve months, and patients were enrolled between February 2023 and November 2024. The local Ethics committee approved the study (PAR 02.23 OSS and 001.23 OSS). All patients gave written informed consent before starting BTx treatment.

Inclusions criteria were age > 18 y; diagnosis of CM according to ICHD-3 (e.g., headache occurring on 15 or more days per month for more than 3 months, of which at least 8 days with headaches either fulfill criteria for migraine) [2]; no previous exposure to BTx; at least one *classic* preventive treatment ongoing at the beginning of the study; and usually stable on preventive treatment for at least 3 months prior to baseline. Exclusion criteria included a history of alcohol or drug abuse, concomitant anti-CGRP therapies, and refusal to sign the consent form.

After patient enrollment, they started at T0 with the first BTx treatment, repeated, as is usual clinical practice, every 3 months for a total duration of twelve months, with careful clinical assessment for ongoing prevention treatments at each visit. Adjustment to concomitant preventive therapies was allowed at the discretion of the headache physician, reflecting usual clinical practice. Study variables were collected at three time points, comprising baseline (T0), T6 (after six months of treatment), and T12 (at the end of the one-year treatment period), and included age, sex, previous preventive failure, baseline preventive treatments, monthly acute medication (MAM), monthly migraine days (MMD), headache impact test (HIT-6) [29], and comorbidities.

### 5.2. Study Endpoints

#### 5.2.1. Primary Study Endpoints

The primary endpoint was to investigate the proportion of patients with chronic migraine who, 12 months after initiation of BTx, achieved a reduction in pharmacological burden of concomitant preventive treatments, defined as discontinuation of one or more previously ongoing preventive medications and maintenance on BTx monotherapy.

#### 5.2.2. Secondary Study Endpoints

- The percentage of patients who, at 12 months, were only on BTx treatment compared to those of patients who were on both BTx and one additional prophylaxis (BTx+1) and to those who were on BTx and two or more additional treatments (BTx ≥ 2);
- The percentage of chronic migraine (CM) patients reverting to episodic migraine (EM) at 6 and 12 months;
- The longitudinal change in monthly acute medication (MAM), monthly migraine days (MMD), and headache impact test (HIT-6) over the course of 6 and 12 months of BTx treatment compared to baseline.

### 5.3. Treatment Protocol

The treatment protocol performed with BTx was based on the PREEMPT protocol [7,9]. We proceeded with 155 units of OnabotulinumtoxinA in 31 fixed-site and fixed-dose injections across seven specific head/neck muscle areas: 5 units in the procero muscle and for each side, 5 units in the corrugator muscles, 10 units in the frontalis muscles, 20 units in the temporalis muscles, 15 units in the occipitalis muscles, 10 units in the cervical paraspinalis muscles, and 15 units in the trapezius muscles. At the investigator’s discretion, with a “follow the pain” approach, an additional 40 units could be administered into the temporalis, occipitalis, and/or trapezius muscles, targeting the side of predominant pain. All injections were performed by headache specialists trained in the PREEMPT protocol.

### 5.4. Methods—Data Analysis

Descriptive analyses were used to report the main features of patients, using frequencies and percentages or means and standard deviations as appropriate.

The primary endpoint was the proportion of patients with CM who, 12 months after initiation of BTx, were on monotherapy; this was reported with a descriptive approach. With regard to secondary endpoints, we addressed the proportion of patients who matched the diagnosis of EM at 6 and 12 months from BTx initiation, and the proportion of patients who reverted to EM, progressed to CM, or were stable in the two categories between 6 and 12 months using descriptive analyses. Parallel to this, we used the non-parametric Friedman’s test to address the variations in the number of preventive treatments other than BTx, MMDs, and MAMs between baseline and 12 months; for this analysis, the non-parametric Wilcoxon’s test was used as a paired post hoc test. Finally, Wilcoxon’s test was also used to address the variation in HIT-6 score between baseline and 12 months; with regard to HIT-6, we also calculated the difference between baseline and 12 months to address the magnitude of change.

The statistical analysis was conducted using SPSS statistic v26.0.

## Figures and Tables

**Table 1:** Demographics, comorbidities, and baseline preventive treatments.

		Patients (*n* = 95)
Demographics	Age (years)	53 ± 11.6
	Female	87
	Male	8
	Previous Failure	3 ± 1.7
Comorbidities		51/95
	Psychiatric traits	20
	Hypertension	14
	Endocrinological disease	11
	Gastrointestinal disorders	9
	Autoimmune disease	4
	Others	18
Baseline Preventive Treatments	Antidepressants	52
	Antiepileptics	30
	Beta blockers	23
	Sartans	17
	Others (Benzodiazepines, myorelaxants, etc.)	13
	Calcium channel antagonist	1

**Table 2:** Frequency and percentage of treatment group, diagnosis, and diagnosis change at T0, T6, and T12.

		T0	T6	T12
Prophylaxis group	BTx only	0 (0%)	17 (17.9%)	27 (28.4%)
	BTx+1	62 (65.3%)	45 (47.4%)	38 (40.0%)
	BTx+2	33 (34.7%)	33 (34.7%)	30 (31.6%)
Diagnosis	EM	0 (0%)	55 (57.9%)	56 (58.9%)
	CM	95 (100%)	40 (42.1%)	39 (41.1%)
Diagnosis Change	Stable CM	–	40 (42.1%)	27 (28.4%)
	From CM to EM	–	55 (57.9%)	13 (13.7%)
	Stable EM	–	–	43 (45.3%)
	From EM to CM	–	–	12 (12.6%)

Notes. BTx = only BTx treatment; BTx+1 = BTx and one additional preventive line; BTx+2 = BTx and two or more additional preventive lines; EM = episodic migraine; CM = chronic migraine.

**Table 3:** Longitudinal changes in MMDs, MAMs, and HIT-6: descriptive statistics and Friedman/Wilcoxon test outcomes.

		T0	T6	T12	Friedman’s Test	Wilcoxon’s Test T0–T6	Wilcoxon’s Test T0–T12
Clinical variables	No. Prophylactic treatments other than BTx (N = 95)	1.5 ± 0.8	1.3 ± 1.0	1.1 ± 1.0	Chi2 = 24.1 *p* < 0.001	z = −2.5 *p* = 0.013	z = −4.4 *p* < 0.001
	MMDs (N = 94)	21.2 ± 6.3	14.3 ± 8.5	13.5 ± 7.6	Chi2 = 71.9 *p* < 0.001	z = −6.6 *p* < 0.001	z = −7.1 *p* < 0.001
	MAMs (N = 84)	22.1 ± 13.4	16.4 ± 16.4	15.8 ± 17.5	Chi2 = 28.1 *p* < 0.001	z = −3.9 *p* < 0.001	z = −4.8 *p* < 0.001
	HIT-6 (N = 90)	66.7 ± 6.3	–	63.8 ± 7.0	–	–	z = −3.7 *p* < 0.001

Notes. BTx = OnabotulinumtoxinA; MMD = monthly migraine days; MAM = monthly acute medications; HIT-6 = headache impact test.

## Data Availability

The original contributions presented in this study are included in the article. Further inquiries can be directed to the corresponding author(s).
